# Feasibility of Virtual Reality Audiological Testing: Prospective Study

**DOI:** 10.2196/26976

**Published:** 2021-08-31

**Authors:** Hye Yoon Seol, Soojin Kang, Jihyun Lim, Sung Hwa Hong, Il Joon Moon

**Affiliations:** 1 Medical Research Institute Sungkyunkwan University School of Medicine Suwon Republic of Korea; 2 Hearing Research Laboratory Samsung Medical Center Seoul Republic of Korea; 3 Center for Clinical Epidemiology Samsung Medical Center Seoul Republic of Korea; 4 Department of Otolaryngology-Head & Neck Surgery Samsung Changwon Hospital Changwon Republic of Korea; 5 Department of Otolaryngology-Head & Neck Surgery Sungkyunkwan University School of Medicine Samsung Medical Center Seoul Republic of Korea

**Keywords:** hearing loss, virtual reality, speech performance, real-world performance, hearing, audiology

## Abstract

**Background:**

It has been noted in the literature that there is a gap between clinical assessment and real-world performance. Real-world conversations entail visual and audio information, yet there are not any audiological assessment tools that include visual information. Virtual reality (VR) technology has been applied to various areas, including audiology. However, the use of VR in speech-in-noise perception has not yet been investigated.

**Objective:**

The purpose of this study was to investigate the impact of virtual space (VS) on speech performance and its feasibility to be used as a speech test instrument. We hypothesized that individuals’ ability to recognize speech would improve when visual cues were provided.

**Methods:**

A total of 30 individuals with normal hearing and 25 individuals with hearing loss completed pure-tone audiometry and the Korean version of the Hearing in Noise Test (K-HINT) under three conditions—conventional K-HINT (cK-HINT), VS on PC (VSPC), and VS head-mounted display (VSHMD)—at –10 dB, –5 dB, 0 dB, and +5 dB signal-to-noise ratios (SNRs). Participants listened to target speech and repeated it back to the tester for all conditions. Hearing aid users in the hearing loss group completed testing under unaided and aided conditions. A questionnaire was administered after testing to gather subjective opinions on the headset, the VSHMD condition, and test preference.

**Results:**

Provision of visual information had a significant impact on speech performance between the normal hearing and hearing impaired groups. The Mann-Whitney *U* test showed statistical significance (*P*<.05) between the two groups under all test conditions. Hearing aid use led to better integration of audio and visual cues. Statistical significance through the Mann-Whitney *U* test was observed for –5 dB (*P*=.04) and 0 dB (*P*=.02) SNRs under the cK-HINT condition, as well as for –10 dB (*P*=.007) and 0 dB (*P*=.04) SNRs under the VSPC condition, between hearing aid and non–hearing aid users. Participants reported positive responses across almost all items on the questionnaire except for the weight of the headset. Participants preferred a test method with visual imagery, but found the headset to be heavy.

**Conclusions:**

Findings are in line with previous literature that showed that visual cues were beneficial for communication. This is the first study to include hearing aid users with a more naturalistic stimulus and a relatively simple test environment, suggesting the feasibility of VR audiological testing in clinical practice.

## Introduction

Hearing loss is a major health concern for the global society due to its negative consequences on individuals’ lives. These consequences include, but are not limited to, communication, employment, cognitive decline, social participation, and quality of life [1-5]. Hearing loss primarily affects communication, and for those who are diagnosed with sensorineural hearing loss, a prescription of hearing aids is typically the first step of the aural rehabilitation process [6]. Hearing aids amplify sounds and provide various features (ie, noise reduction) to substantially mitigate the negative consequences of hearing loss by improving audibility. However, even with these advancements, there is a gap between clinical assessment and real-world performance [7-13], such as the wearer’s complaint of persistent hearing difficulties in noisy situations [14-17].

One contributing factor for this issue could be limitations of current measurement tools. Taylor [18] mentioned difficulties in constructing a laboratory environment that closely replicates real-world settings and in measuring individuals’ unique auditory environments. In clinical practice, aided threshold and speech perception testing is often performed to assess the benefits provided by hearing aids. Aided threshold testing involves presenting warbles tones (250 Hz to 8000 Hz) through a loudspeaker in the sound field [19]. The patient is asked to respond (ie, “say *yes* or press the button”) when he or she hears the tone, even if the tone is soft. Speech testing is also performed in the sound field with one or more loudspeakers [19]. Words and sentences can be used as test materials and the patient is asked to listen and repeat words and sentences back to the tester. Some outcome measures include noise and multi-talker conditions to simulate real-world auditory environments, but they lack an important piece of information that people use for communication: visual cues. In real-world conversations, nonverbal cues, such as lip movements, are readily available and their significant influence on speech perception has been demonstrated in previous studies [20-25]. Summerfield [21] examined changes in the accuracy of phonetic perception in noise depending on the amount of visual information given to 10 listeners with normal hearing (NH). The participants heard a total of 125 sentences with 100 keywords and repeated the keywords under various conditions. Participants’ overall speech performance was the best when they were able to see the whole face of the speaker (65.3%), followed by the lips (54.0%), the four points (30.7%), nothing (22.7%), and a circle (20.8%). A more recent study investigated the speech perception performance of 77 NH participants who were divided into five age groups under three conditions: auditory only, visual only, and audiovisual. The highest accuracy rate was observed for the audiovisual condition, followed by the auditory-only and the visual-only conditions [26]. Benefits of audiovisual integration are well noted in literature and, yet, there are no audiological evaluation tools that use visual cues. Thus, even with well-programmed devices and good test results, hearing aid wearers often do not perceive this benefit in the real world. This mismatch reduces device satisfaction and can ultimately result in discontinuance of hearing aid use [7-9,11-13,27,28]. The MarkeTrak survey conducted in 2000 by Sergei Kochkin reported poor benefit as one of the top 10 reasons for not using hearing aids [27]. Results from previous studies emphasize the need for closing the gap between clinical assessment and real-world performance.

With the emergence of the fourth industrial revolution, researchers and industries have been putting in efforts to fuse technologies and health care. Among these technologies, virtual reality (VR) has been applied not only to gaming but to education and health care. There are five key components of VR systems: virtual space (VS), immersion, interactivity, creators, and users [29]. To be more specific, there is an imaginary place, VS, and through interaction and immersion, individuals feel more present, or connected, to the VS. VR’s biggest strength is that auditory and visual information are provided simultaneously to generate realistic environments. There are studies showing the efficacy of VR in certain areas, such as pain management, stroke rehabilitation, and chronic subjective tinnitus [30-34]. For audiology, VR has been researched for sound localization, but research into the effect of VS on speech performance has been sparse [35-37].

Ahrens et al [37] tested the sound localization ability of 10 NH listeners under eight conditions involving blindfolding, a head-mounted display (HMD) headset, virtual and real environments, loudspeakers, acoustic or visual stimuli, and a simulated laser pointer. The results revealed that the headset had a negative impact on individuals’ sound localization ability, as differences in interaural time and levels, which are important cues for sound localization, were larger when wearing the headset. Azimuth and elevation errors decreased when the source locations were visible to the participants in both the virtual and real environments. Sechler et al [38] explored the potential use of VR in sound localization testing among bilateral cochlear implant users. A total of 12 NH listeners and four bilateral cochlear implant users performed sound localization testing in a VR environment that was created for the study. A total of 13 sound cues were presented and the participants selected where they heard the sound cues in the VR environment. Bilateral cochlear implant users completed testing under first implant–only, second implant–only, and both implants conditions. Comparing the localization performance, individuals with NH showed better performance than bilateral cochlear implant users as to response time, left or right discrimination, percent correct, and root mean square error. Better sound localization performance was observed under the implants condition and the first implant–only condition. Overall, both studies suggest VR’s potential to be employed in clinical audiology.

The purpose of this study was to investigate the impact of VS on speech-in-noise performance and its feasibility as a viable instrument for speech testing in clinical practice. Findings from this study will shed light on VR’s potential to overcome the limitations of current assessment tools and, ultimately, to be utilized in clinical practice, which is an unexplored territory in the field of audiology.

## Methods

### Participants

**Figure 1:**
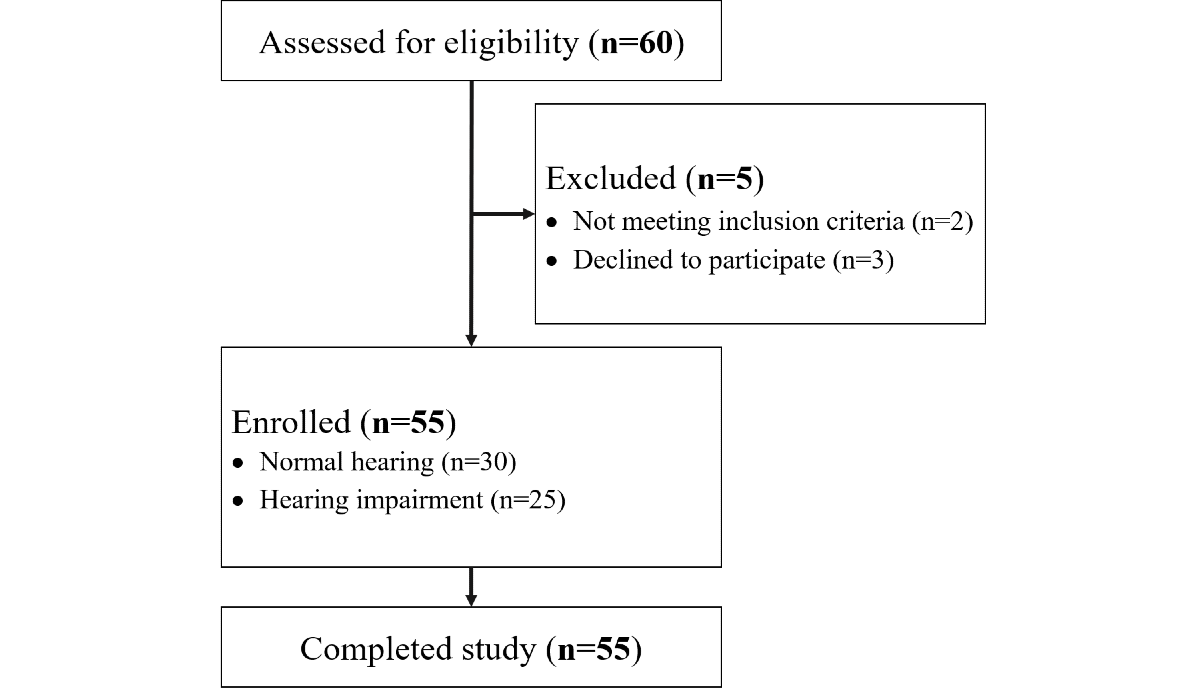
CONSORT (Consolidated Standards of Reporting Trials) diagram of study participation.

The sample size was determined based on previous research examining reaction time and accuracy differences under auditory-only and visual-only conditions among individuals with and without autism [39]. The resulting sample size was 45, using Stata version 14 (StataCorp LP) for power set at 0.9 and α set at .0167 (corrected for multiple comparison). A total of 30 individuals with NH and 25 hearing impaired (HI) individuals were enrolled in the study (Figure 1). The NH group had average pure-tone thresholds below 25 dB hearing level (HL), with an asymmetry in hearing thresholds below 10 dB across 250 Hz, 500 Hz, 1000 Hz, 2000 Hz, 4000 Hz, and 8000 Hz. The HI group had average pure-tone thresholds above 25 dB HL, with an asymmetry in hearing thresholds below 10 dB across the testing frequencies. For the HI group, 10 individuals were hearing aid users. Individuals who were unable to communicate and understand television at a distance of 1 meter and those with neurological and mental disorders were excluded from the study. All experimental procedures were approved by the regulations set by Samsung Medical Center’s Institutional Review Board and were carried out in accordance with approved guidelines. An informed consent document was obtained prior to testing from the participants. Informed consent was also obtained from actors to publish the images in an online publication.

### Conventional Pure-Tone Audiometry

Following the 2005 American Speech-Language-Hearing Association guidelines [40], conventional pure-tone audiometry was performed in a sound booth using a GSI 61 audiometer (Grason-Stadler) and TDH-39 headphones (Telephonics).

### Virtual Space

**Figure 2:**
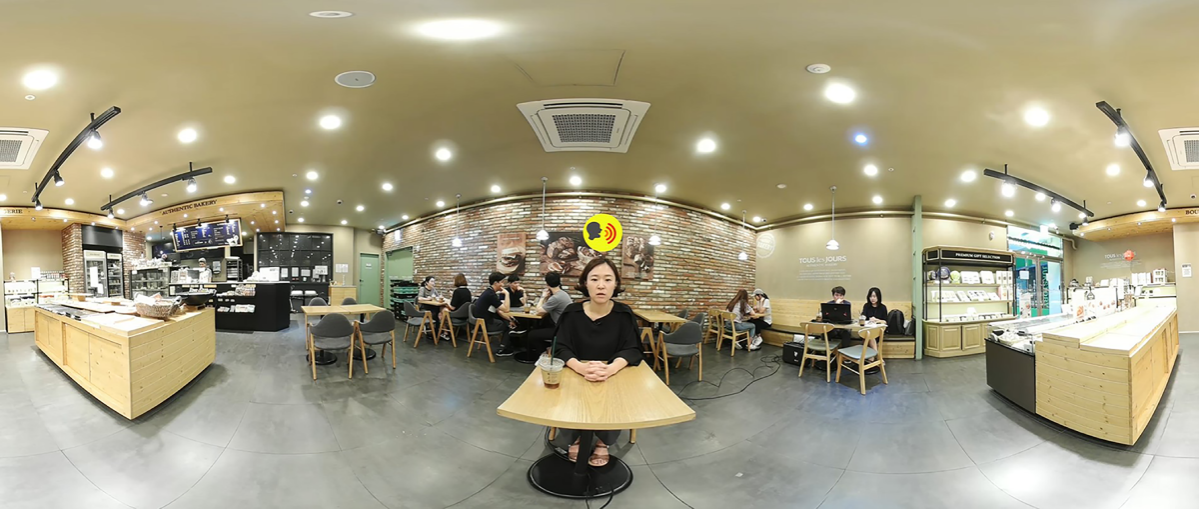
A screenshot of the virtual space. The “conversational partner” is speaking sentences from the Korean version of the Hearing in Noise Test (K-HINT), while the “customers” are talking in the background.

A café was created as a VS with the assistance of the Samsung Changwon Hospital VR Lab using the Samsung 360 Round VR camera (Samsung Electronics Co). The film was then edited using commercial editing tools from Adobe Systems: Adobe Premiere Pro, Adobe After Effects, and Adobe Audition (2018-2019 versions). A café was selected as an environment as it is one of the most common places within which individuals have trouble hearing [41,42]. A scenario for the VS where the user is having a one-on-one conversation with a conversational partner who speaks sentences from the Korean version of the Hearing in Noise Test (K-HINT), while other “customers” are talking in the background, was designed (Figure 2). The conversational partner recorded the K-HINT sentences.

### K-HINT

The K-HINT, developed by Sung Kyun Moon and his colleagues at Ajou University and the House Ear Institute, is widely used in Korea as a speech-in-noise test [43], with a listen-and-repeat task. The K-HINT consists of 12 lists with 20 sentences per list. The K-HINT sentences were used for the study with a presentation level of 65 dBA (A-weighted dB). Each list was broken down into two sets in order for the participants to complete all test conditions: (1) conventional K-HINT (cK-HINT); (2) VS on PC (VSPC), where the VS was displayed on a monitor; and (3) VS head-mounted display (VSHMD), where the VS was displayed on the HMD at –10, –5, 0, and +5 dB signal-to-noise ratios (SNRs). For VSPC and VSHMD conditions, the same VS was displayed and all participants had 10 seconds to familiarize themselves with the virtual environment. The test conditions were randomized for each participant. Percent-correct scores were calculated based on the number of sentences that were repeated back to the tester correctly among 10 sentences. The hearing aid wearers used their own hearing aids to complete testing under unaided and aided conditions. No adjustments were made to the participants’ hearing aid settings, as the authors wanted to simulate as natural an environment as possible. Testing was performed in a semianechoic chamber with sentences being presented through a loudspeaker in the front. Making the testing more realistic, café noise was obtained from YouTube and normalized to the average level of the sound file using Cool Edit Pro 2.1 (Syntrillium Software Corporation). Then, sound levels of speech as well as the café noise were measured using a sound level meter for the four SNRs. The noise was presented from speakers located at 45, 135, 225, and 315 degrees for all test conditions. A Samsung Notebook Odyssey laptop and a Samsung Odyssey VR headset with controllers (Samsung Electronics Co) were used to display the VS. The Samsung Odyssey laptop was used, as testers can see the screen that participants are seeing during the VSHMD condition. This allows individuals who are unfamiliar with VR technology to easily complete the task with assistance from the tester. A practice test was run before the experiment to familiarize the participants with the listen-and-repeat task in the VS.

### Questionnaire

A questionnaire was administered after testing to evaluate various aspects of testing (Table 1). The questionnaire contained four domains: HMD headset, VSHMD condition, tests, and cK-HINT versus VSHMD. Items regarding the headset consisted of physical comfort and weight of the device, audiovisual synchronization, and sound quality of the recorded K-HINT sentences. In terms of the VSHMD condition, immersiveness, listening effort, degree of reality reflection, need for VR to be incorporated into audiological testing, adequacy of the VS, structure of the test, and interestingness were evaluated. For questions about immersiveness and listening effort, hearing aid users completed these questions twice for unaided and aided conditions. Participants also chose the most preferred test method and the test that required the greatest amount of listening effort, encouraged hearing aid use, and assessed communication difficulties better in the *tests* domain. The last domain compared the cK-HINT and VSHMD conditions. Participants were asked to write down strengths and weaknesses of the two conditions. In terms of differences between the two conditions, participants had an option to choose multiple responses among *immersiveness*, *reality reflection*, *convenience*, and *others*. If they chose *others*, they were asked to provide specific responses. Questions in the *HMD headset* and *VSHMD condition* domains were answered using the 10-point Visual Analogue Scale (VAS) with the following respones: 0 (poor, strongly disagree, or extremely heavy), 5 (neutral), and 10 (excellent, strongly agree, or extremely light). The *tests* domain contained multiple-choice questions. Participants had to choose from the following response options: *cK-HINT*, *VSPC*, and *VSHMD*.

**Table 1:** Questionnaire items.

Domain and question No.		Question
**HMD^a^ headset**		
	1	How is the physical comfort of the device?
	2	How heavy is the device?
	3	Does the visualization (café) match well with the audio?
	4	How is the sound quality?
**VSHMD^b^ condition**		
	5	Without hearing aids: How immersive was the virtual space? Did you feel like you were in a real café?
	5-1	With hearing aids: How immersive was the virtual space? Did you feel like you were in a real café?
	6	Without hearing aids: How much effort did you have to spend to understand speech?
	6-1	With hearing aids: How much effort did you have to spend to understand speech?
	7	How much did the virtual space (café) reflect reality?
	8	Does the virtual reality technology need to be used for clinical testing?
	9	Was the café an appropriate place to use as the virtual space?
	10	Was the test structured to be easily understood?
	11	Was the test interesting?
**Tests**		
	12	Which test do you prefer the most?
	13	Which test required the most amount of effort for listening?
	14	Which test would encourage hearing aid use?
	15	Which test would assess communication difficulties better?
**cK-HINT^c^ versus VSHMD condition**		
	16	Describe any differences between conventional testing (without visual cues) and the VSHMD (visualization through the headset).
	17	Describe strengths and weaknesses of conventional testing (without visual cues) and the VSHMD (visualization through the headset).

^a^HMD: head-mounted display.

^b^VSHMD: virtual space head-mounted display.

^c^cK-HINT: conventional Korean version of the Hearing in Noise Test.

### Statistical Analysis

Statistical analysis was performed using SAS version 9.4 (SAS Institute Inc). Nonparametric tests were used, as our results did not pass the normality test. To compare K-HINT performance based on test conditions in each group and SNRs, the Friedman test was performed. The primary outcome was individuals’ K-HINT performance, and the availability of visual cues was the exposure of interest in this study. To compare K-HINT performance between the groups, the Mann-Whitney *U* test was used. A *P* value of less than .05 was considered to be statistically significant.

## Results

### Participant Characteristics

The age range of the participants was 18 to 75 years old. The mean age of the NH group was 29.7 years (SD 10.4), while the mean age of the HI group was 53.0 years (SD 14.0). The NH group’s pure-tone averages were 6.3 dB in the right ear and 5.5 dB in the left ear. The HI group had pure-tone averages of 49.2 dB in the right ear and 47.2 dB in the left ear. A total of 10 participants in the HI group were hearing aid users, with pure-tone averages of 56.5 dB and 54.2 dB in the right and left ears, respectively. Non–hearing aid users in the HI group had pure-tone averages of 37.0 dB and 34.9 dB in the right and left ears, respectively.

### K-HINT Performance Between the NH and HI Groups

**Figure 3:**
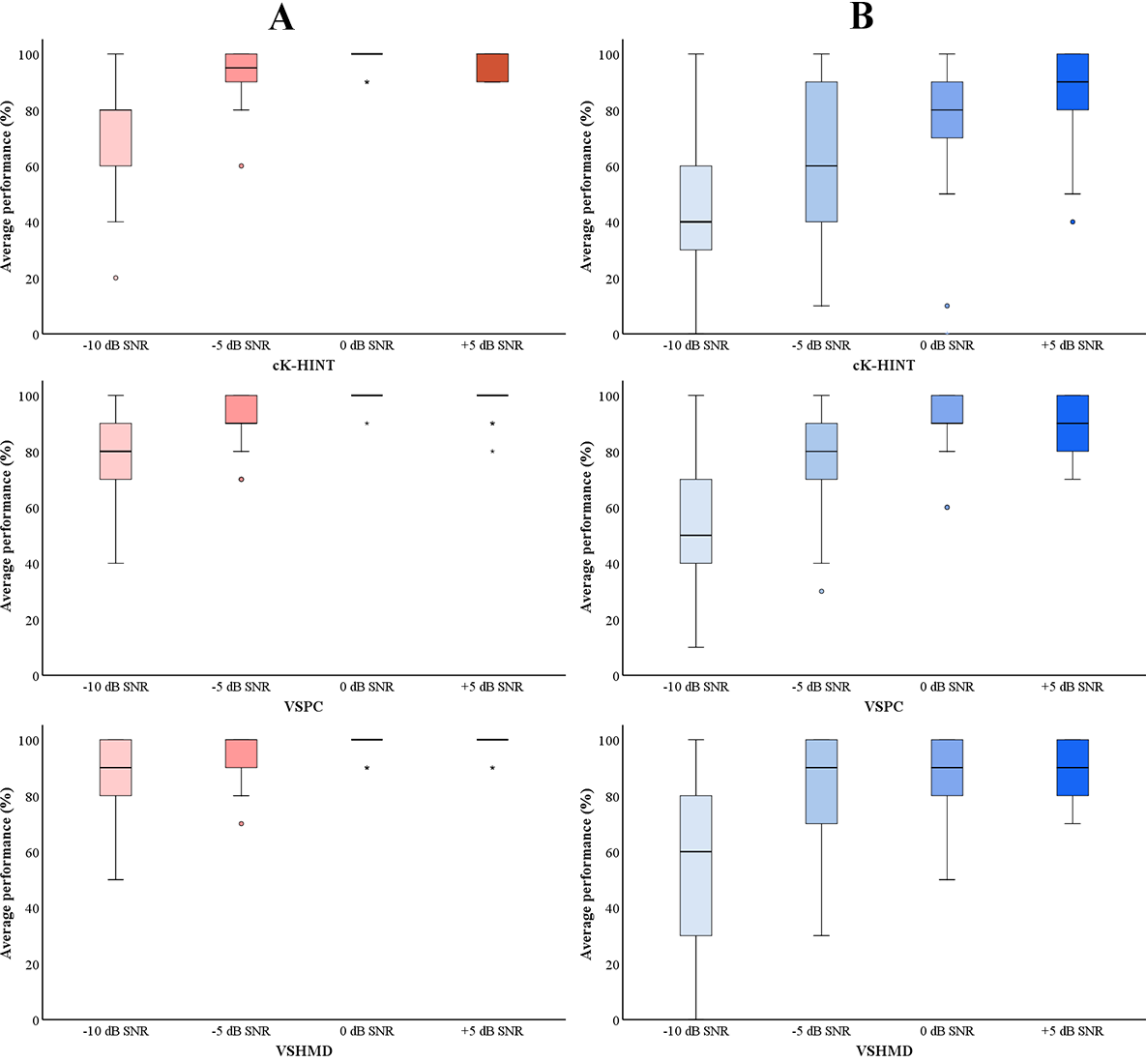
Statistical analysis of the groups’ average speech performance. Pink bars (A) indicate normal hearing group’s performance. Blue bars (B) indicate hearing impaired group’s performance. The horizontal lines within the shaded bars represent the median values, the shaded bars represent the IQRs, the error whiskers represent the highest and lowest points, and the circles and stars represent outliers and extreme outliers, respectively. cK-HINT: conventional Korean version of the Hearing in Noise Test; SNR: signal-to-noise ratio; VSHMD: virtual space head-mounted display; VSPC: virtual space on PC.

Comparison of the K-HINT performance of both groups under all test conditions is illustrated in Figure 3. For hearing aid users, their percent-correct scores for the aided conditions were used for comparison. Both groups performed better when visual cues were available. The Friedman test was performed for each group to examine whether provision of visual signals was beneficial. Statistical significance was observed for –10 dB SNR in the NH group (*P*=.004) and for –10 dB (*P*=.01), –5 dB (*P*=.01), and 0 dB (*P*=.045) SNRs in the HI group. Group comparison using the Mann-Whitney *U* test showed statistical significance (*P*<.05) between the two groups under all test conditions, with *P* values ranging from .001 to .004. Overall, NH listeners showed better speech-in-noise performance than the HI group.

### VR K-HINT Performance Between Hearing Aid Users and Non–Hearing Aid Users in the HI Group

**Figure 4:**
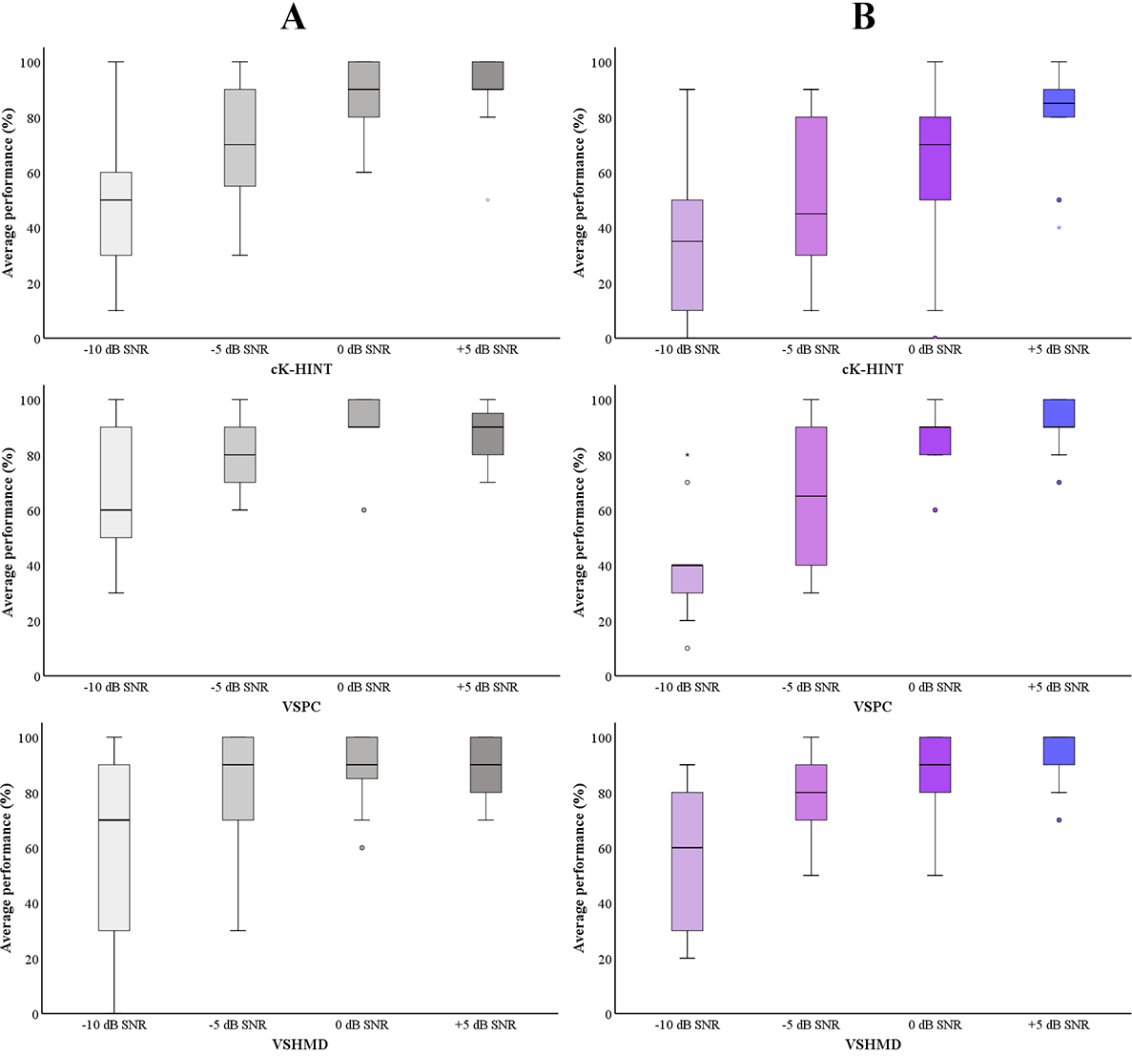
Statistical analysis of the groups’ average speech performance. Gray bars (A) indicate non–hearing aid users’ performance. Purple bars (B) indicate hearing aid users’ performance. The horizontal lines within the shaded bars represent the median values, the shaded bars represent IQRs, the error whiskers represent the highest and lowest points, and the circles and stars represent outliers and extreme outliers, respectively. cK-HINT: conventional Korean version of the Hearing in Noise Test; SNR: signal-to-noise ratio; VSHMD: virtual space head-mounted display; VSPC: virtual space on PC.

The K-HINT performance of hearing aid and non–hearing aid users is shown in Figure 4. Hearing aid users’ aided scores were used for performance comparison. Higher average percent-correct scores for non–hearing aid users indicate that they understood speech better in noise than hearing aid users. This is consistent with non–hearing aid users’ and hearing aid users’ pure-tone audiometry data: non–hearing aid users had better audiometric thresholds across the testing frequencies, except at 4000 Hz and 8000 Hz in the left ear. The Mann-Whitney *U* test revealed statistical significance at –5 dB (*P*=.04) and 0 dB (*P*=.02) SNRs under the cK-HINT condition and at –10 dB (*P*=.007) and 0 dB (*P*=.04) SNRs under the VSPC condition between the non–hearing aid users and hearing aid users.

### Hearing Aid Users’ Unaided and Aided VR K-HINT Performance

**Figure 5:**
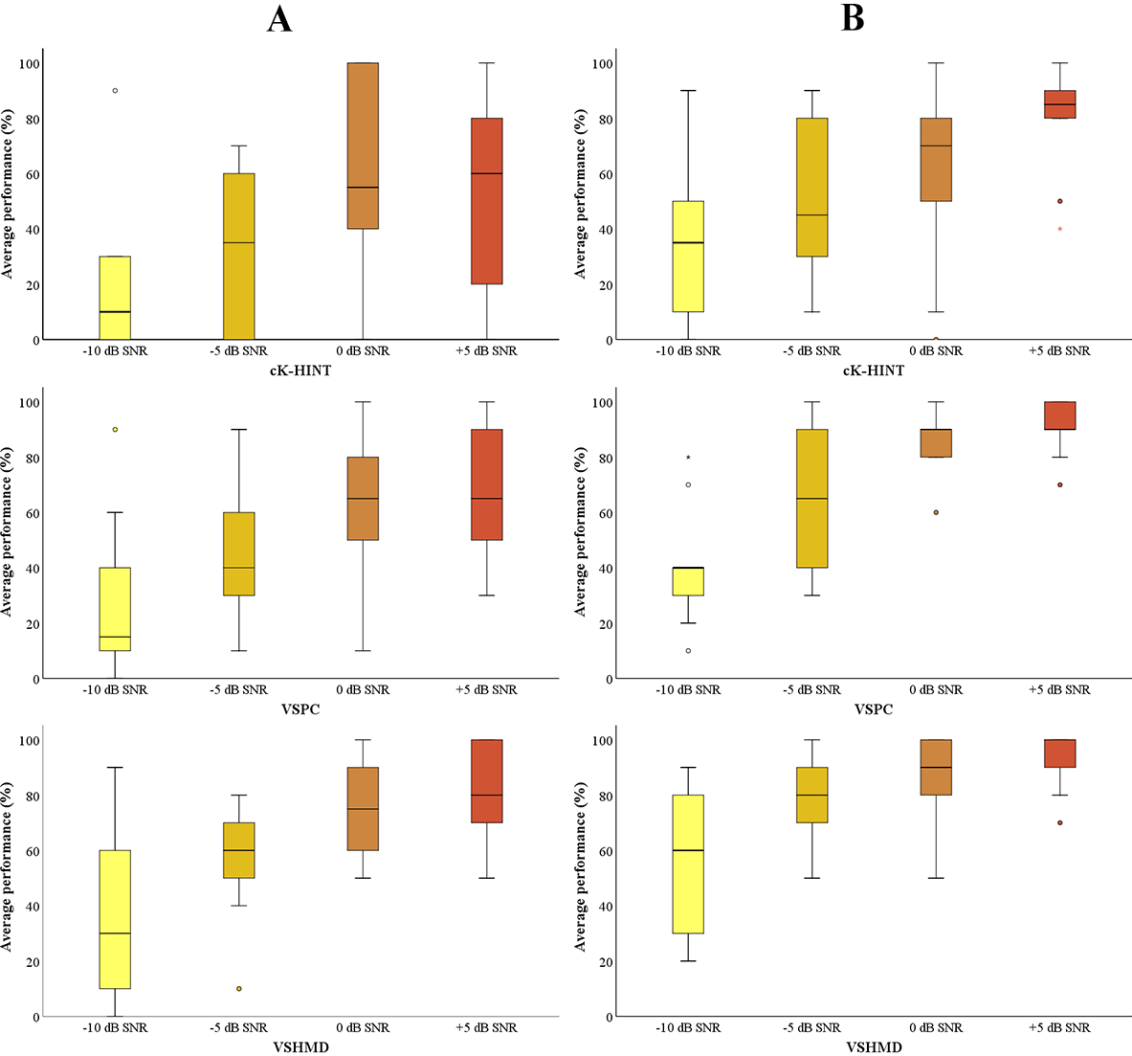
Statistical analysis of hearing aid users’ average performance of the Korean version of the Hearing in Noise Test (K-HINT) in unaided (A) and aided (B) conditions. The horizontal lines within the shaded bars represent the median values, the shaded bars represent the IQRs, the error whiskers represent the highest and lowest points, and the circles and stars represent outliers and extreme outliers, respectively. cK-HINT: conventional Korean version of the Hearing in Noise Test; SNR: signal-to-noise ratio; VSHMD: virtual space head-mounted display; VSPC: virtual space on PC.

Figure 5 displays hearing aid users’ K-HINT performance with and without their hearing aids. The results are in line with previous studies showing that speech-understanding-in-noise performance is better with hearing aids. Statistical significance was also observed for +5 dB SNR under the cK-HINT condition (*P*=.02); –10 dB (*P*=.04), –5 dB (*P*=.02), and +5 dB (*P*=.02) SNRs under the VSPC condition; and –10 dB (*P*=.04) and –5 dB (*P*=.002) SNRs under the VSHMD condition through the Wilcoxon signed-rank test.

### Questionnaire

**Figure 6:**
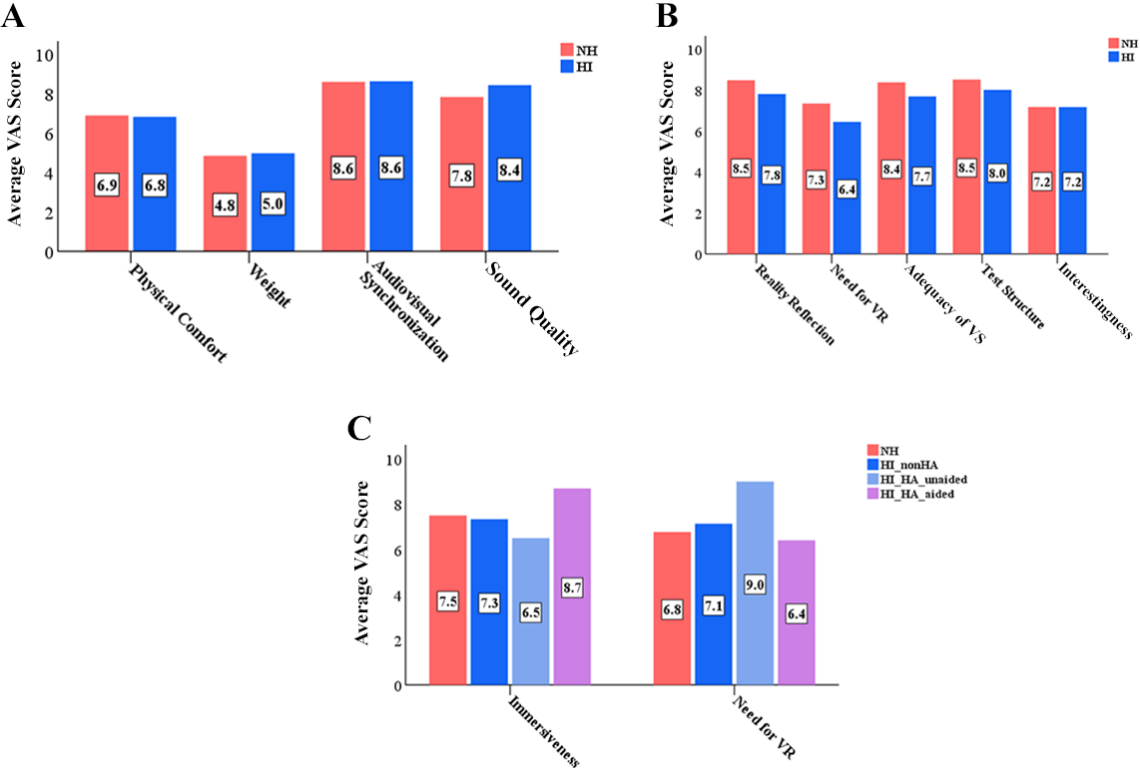
(A) Questionnaire results regarding the head-mounted display (HMD) headset. Pink bars represent average responses from the normal hearing (NH) group and blue bars represent average responses from the hearing impaired (HI) group. A value of zero (0) on the Visual Analogue Scale (VAS) indicates *extremely heavy* or *poor*, while 5 indicates *neutral* and 1 indicates *extremely light* or *excellent*. (B) Questionnaire results for the virtual space head-mounted display (VSHMD) condition. Pink bars represent average responses from the NH group and blue bars represent average responses from the HI group. (C) Questionnaire results for the VSHMD condition regarding the amount of perceptual presentation in the virtual space (VS) and effort exerted to understand speech in noise. Pink bars indicate average responses from the NH group, while blue, sky blue, and purple bars indicate average responses from non–hearing aid (nonHA) users, hearing aid (HA) users in the unaided condition, and hearing aid users in the aided condition, respectively. VR: virtual reality.

The groups’ subjective opinions on the headset, the VSHMD condition, listening effort, and presence in the VS were gathered through a questionnaire (Figure 6). For the HMD headset, the following items were evaluated: physical comfort when wearing the device, weight of the device, synchronization between audio and visual information, and sound quality of the recorded sentences. The VAS was used to rate the items, with 10 being *strongly agree* or *excellent*. Responses for all items, except for the weight of the device, were positive toward the system; the headset was heavy, but was comfortable to wear and had excellent sound quality and audiovisual synchronization. The degree of reality reflection, need for VR to be used in clinical testing, adequacy of VS, test structure, and interestingness regarding the VSHMD condition were also evaluated. The VAS was used to rate the items, with 10 being *strongly agree* or *excellent*. The results revealed that reality simulation through the VS was excellent, and participants felt that the testing was interesting. The café was an appropriate place to use as the VS. The test structure, in which participants completed practice runs and then experimental tests, was considered good as well. The necessity of VR in audiology was high. Lastly, immersion and listening effort were investigated. Since the hearing aid users completed testing under unaided and aided conditions, the amount of listening effort required for these conditions was evaluated twice. Responses from NH listeners and non–hearing aid users were similar to each other across all items; the Wilcoxon signed-rank test showed no statistical differences for immersion (*P*=.36) and listening effort (*P*=.49) for the NH and HI groups. For hearing aid users, scores were higher for immersion and lower for listening effort with hearing aids, implying that integration of auditory and visual information through hearing aids and visual cues have a positive impact on speech understanding in the presence of noise. Significant differences for immersion (*P*=.047) and listening effort (*P*=.04) between the unaided and aided conditions were also observed through the Wilcoxon signed-rank test.

Both groups preferred tests that contained visual cues; 50% (15/30) and 32% (8/25) of the participants in the NH and HI groups, respectively, selected VSHMD. VSHMD was also selected the most by the groups as a test that better-assessed communication difficulties (67% [20/30] of the NH group and 52% [13/25] of the HI group) and encouraged hearing aid use (50% [15/30] of the NH group and 44% [11/25] of the HI group). The cK-HINT, which did not provide any visual information, required the greatest amount of listening effort, as reported by the NH (22/30, 73%) and HI (20/25, 80%) groups. A total of 97% (29/30) of participants in the NH group and 88% (22/25) of participants in the HI group showed willingness to complete the test if available in clinical practice.

### Differences Between the cK-HINT and VSHMD Conditions

Simulation of reality and immersion were the main differences reported by the groups. For this question, individuals were able to select more than one option. A total of 63% (19/30) and 83% (25/30) of participants in the NH group selected immersion and simulation of a real-world environment, respectively, as differences between the conditions. For the HI group, 72% (18/25) and 52% (13/25) of participants selected immersion and reality simulation, respectively, as differences between the conditions. Other responses included “test is interesting,” “being able to concentrate during testing,” and “visual cues (ie, lip movements) were available.” This adds value to VR’s strengths and participants’ subjective responses regarding test preference and tests that better promote hearing aid use and assess hearing problems.

### Strengths and Weaknesses of the cK-HINT and VSHMD Conditions

A substantial majority of the participants reported that the cK-HINT would be a better assessment tool for measuring auditory performance because it did not provide any visual cues: they only had auditory information to understand speech. Convenience of testing was another strength of the condition, as it did not require additional devices for the participants to wear. Weaknesses of the condition, on the other hand, included boredom, no provision of visual information, unrealistic testing environment, and noise. Since no visual cues were presented as part of this condition, participants thought that the test would not be able to accurately assess their real-world speech-in-noise performance. For the VSHMD condition, both groups reported the following strengths: less effort to hear, excellent audio and visual quality, excellent reality reflection, feeling present in the environment, and increased concentration during testing. Utilizing visual cues during testing helped the participants exert less effort to understand speech in the presence of noise. Excellent audio and visual quality and reality reflection allowed them to feel present in the VS during testing. However, the headset was heavy to wear, which the participants thought could possibly affect the test results. In terms of weakness, they mentioned that individuals who are not familiar with the HMD system might have difficulty performing the test (ie, wearing the device and navigating through the test) and that visualization provided as part of this condition might distract individuals.

## Discussion

In our study, speech recognition improved with the provision of visual information, regardless of the presence of hearing loss. Hearing aids facilitated better speech recognition with lower listening effort for hearing aid wearers. All of these findings are consistent with previous literature [44-49]. After summarizing participants’ subjective responses, we saw that the quality of the VS was excellent, which was demonstrated by high scores for audiovisual synchronization, audio quality of the recorded sentences, immersion, and the amount of reality reflected in the VS. A café was an appropriate place to use as the VS. Participants were interested in the new testing method (ie, VSHMD), and a high percentage of participants showed inclination toward completing the test once available in clinic.

Our study is meaningful in terms of diversity of participant characteristics, a relatively simple test environment, and a more naturalistic stimulus. Most studies utilizing VR for speech performance recruited individuals with NH and HI [36,50-52] and involved a test setup that may be difficult to establish in clinical settings. For example, in Salanger et al [50], 40 children and 8 young adults with NH were enrolled in the study. Acoustic treatments (ie, acoustic wall and ceiling tiles) and objects (ie, chalkboards) were included to create a VR 3D classroom. Video recordings of the talkers, which were less naturalistic, were presented to the participants. Hendrikse et al [51] also recruited 14 young NH listeners for localization and speech performance testing with animated characters as test stimuli. A 16-loudspeaker array and a projector were used to present auditory and visual stimuli, and a metal frame covered by a cloth was used to reduce environmental sounds, light, and room reflections [51]. Setting up such test environments in clinical practice may be challenging, as they require a number of loudspeakers, large space, and other necessary materials for the creation of a realistic environment. Our study, on the other hand, is the first study to include hearing aid users with a more naturalistic stimulus and a potentially more implementable test setup for clinical practice. The VS presented in this study was created using a real café and actors instead of avatars. The testing was performed using a relatively fewer number of loudspeakers (five) and, yet, the results were comparable to that obtained in previous studies.

Incorporation of visual information into speech testing can be beneficial for both patients and professionals. Since communication entails visual and auditory information, this type of testing could assess communication difficulties in conjunction with speech recognition performance more accurately. Patients would be more engaged in testing since the test is more interesting, as reported on the questionnaire. For hearing aid users, reality-reflected test results could foster realistic expectations, ownership of hearing loss, and better optimization of the devices. This would lead to increased satisfaction toward the device and reduced hearing aid return and discontinuance rate. If hearing aid wearers experience higher device satisfaction and perceived hearing aid benefit, the number of clinic visits for further adjustments would also decrease, which can be a critical issue for individuals who live far away from hospitals and clinics.

Although a VS is shown to be beneficial for speech recognition in noise in this study, ample work is still needed to address some limitations of our research. Each K-HINT list was broken down into two separate lists so that hearing aid users could complete tests under unaided and aided conditions. It is highly likely that phoneme distribution was affected during this process and, therefore, test materials with more sentence lists need to be used for subsequent studies. The weight of the headset also needs to be improved. The authors believe that it is crucial to not only examine the effect of visual cues on speech performance but to test the device that will be used, as it could be one of many factors that professionals and patients would consider before employing and performing the test in clinics. The weight of the system was reported to be heavy on the questionnaire and was mentioned as a weakness of the VSHMD condition. Use of a lighter device could possibly address this concern. Another concern was that individuals who are unfamiliar with the HMD system might have difficulty performing the test. Designing user interfaces that are easy to use and providing tester assistance regarding the HMD system before and during testing could address the issue. It is also worth mentioning that in-depth investigation as to the amount contributed by each sensory modality for speech-in-noise performance is necessary. Gonzalez-Franco et al [53] examined the impact of selective attention on individuals’ speech perception when visual and auditory cues were asynchronous. Two speakers were simultaneously speaking sentences, and participants were asked to recall the “target CALL,” which consisted of eight words (“Arrow,” “Baron,” etc), and to remember the content of the target sentences under four conditions (ie, synchronized visual and audio cues; auditory only with no visual information; asynchronized visual and audio cues, in which the target speaker’s lips matched the audio of the other talker; and asynchronized visual and audio cues, in which the target speaker’s lips did not match any talker’s audio). Participants were able to identify the “target CALL” more accurately when auditory and visual information was synchronous. In terms of remembering the content of the sentences, more errors were observed with asynchronous information, especially when the target speaker’s lips matched the audio of the other speaker, demonstrating the dominance of visual cues [53]. Measuring one’s reliance on each sensory system might allow researchers to recognize whether a test containing visual cues reflects one’s speech performance in a real environment; if one’s communication is actually interfered with by visual signals occurring naturally in real life and the test scores are poor, this might mean that the test is reflective of his or her real-world performance. In addition, vision screening was not performed prior to testing. Although the authors made sure all participants were able to clearly see the VS for the VSHMD condition, as the rationale behind the experiment is visual and auditory input representing real-world conditions, it is necessary to include vision screening. There is a possibility of different hearing aid settings affecting the HI group’s speech performance. As mentioned earlier, the authors did not make any changes to the hearing aid settings because those are the settings that are used by hearing aid users in the real world. However, some features, such as noise reduction, might have influenced the results of the HI group. It is worth noting that in-depth investigations regarding the actual impact of VR audiological testing in clinical practice is necessary. For example, the Technology Acceptance Model is commonly used for implementation-focused research to examine user acceptance of information technology by evaluating individuals’ willingness to use technology, perceived ease of use, and so on [54]. It is important to not only compare performance but also assess end-user acceptance of VR audiological testing to fully understand how VR audiological testing works and compares to other testing methods. Further studies with larger sample sizes, a larger variety of participant characteristics, and correlational analysis between speech performance with visual cues and standardized hearing aid questionnaires would be beneficial. Development of sentences that are appropriate for the VS and examination of their effect would be valuable in taking a step forward toward the development and standardization of reality-reflecting test methods and materials. In sum, we hope our findings open up opportunities for future studies and support the necessity of VR in being utilized in the field of audiology. It might still be challenging to set up a test environment that closely resembles individuals’ everyday listening environments and to accurately evaluate one’s unique hearing difficulties and needs. However, VR audiological testing would be another way for professionals to serve diverse clinical populations more competently.

## Abbreviations

cK-HINT: conventional Korean version of the Hearing in Noise Test
dBA: A-weighted dB
HI: hearing impaired
HL: hearing level
HMD: head-mounted display
K-HINT: Korean version of the Hearing in Noise Test
NH: normal hearing
NRF: National Research Foundation of Korea
SNR: signal-to-noise ratio
VAS: Visual Analogue Scale
VR: virtual reality
VS: virtual space
VSHMD: virtual space head-mounted display
VSPC: virtual space on PC
