# Correction: Feasibility of Virtual Reality Audiological Testing: Prospective Study

**DOI:** 10.2196/34994

**Published:** 2021-11-25

**Authors:** Hye Yoon Seol, Soojin Kang, Jihyun Lim, Sung Hwa Hong, Il Joon Moon

**Affiliations:** 1 Medical Research Institute Sungkyunkwan University School of Medicine Suwon Republic of Korea; 2 Hearing Research Laboratory Samsung Medical Center Seoul Republic of Korea; 3 Center for Clinical Epidemiology Samsung Medical Center Seoul Republic of Korea; 4 Department of Otolaryngology-Head & Neck Surgery Samsung Changwon Hospital Changwon Republic of Korea; 5 Department of Otolaryngology-Head & Neck Surgery Sungkyunkwan University School of Medicine Samsung Medical Center Seoul Republic of Korea

In “Feasibility of Virtual Reality Audiological Testing: Prospective Study” (JMIR Serious Games 2021;9(3):e26976) one error was noted.

In the originally published paper, author affiliation 5, applying to Corresponding Author Il Joon Moon, was as follows:

Department of Otolaryngology-Head & Neck Surgery, Samsung Medical Center, Seoul, Republic of Korea

This has been corrected to:

Department of Otolaryngology-Head & Neck Surgery, Sungkyunkwan University School of Medicine, Samsung Medical Center, Seoul, Republic of Korea

Accordingly, the Corresponding Author address for Il Joon Moon has also been corrected from:

Il Joon Moon, MD, PhD

Department of Otolaryngology-Head & Neck Surgery

Samsung Medical Center

81 Irwon-ro, Gangnam-gu

Seoul, 06351

Republic of Korea

Phone: 82 2 3410 3579

Email: moonij@skku.edu

to:

Il Joon Moon, MD, PhD

Department of Otolaryngology-Head & Neck Surgery

Sungkyunkwan University School of Medicine

Samsung Medical Center

81 Irwon-ro, Gangnam-gu

Seoul, 06351

Republic of Korea

Phone: 82 2 3410 3579

Email: moonij@skku.edu

The correction will appear in the online version of the paper on the JMIR Publications website on November 25, 2021, together with the publication of this correction notice. Because this was made after submission to PubMed, PubMed Central, and other full-text repositories, the corrected article has also been resubmitted to those repositories.

